# Factors influencing the mental health of young people with disability in Australia: a systematic scoping review

**DOI:** 10.1186/s12889-026-28583-8

**Published:** 2026-07-22

**Authors:** Jodie Bailie, Zoe Aitken, Stephanie Mantilla, Marissa Shields, Stefanie Dimov, Tess Bright, Alex Sully, Noah Folpp, Kanchana Ekanayake, Gwynnyth Llewellyn

**Affiliations:** 1 Centre for Disability Research and Policy, The University of Sydney, Camperdown, NSW Australia; 2 University Centre for Rural Health, The University of Sydney, 61 Uralba Street, Lismore, NSW 2480 Australia; 3 Melbourne School of Population and Global Health, University of Melbourne, Melbourne, VIC Australia; 4 Sydney Medical School, The University of Sydney, Camperdown, NSW Australia; 5 University of Sydney Library, The University of Sydney, Camperdown, Australia

**Keywords:** Mental health, Disability, Youth, Socio-ecological model, Review

## Abstract

**Objectives:**

Young Australians with disability experience poorer mental health than their non-disabled peers. Despite this, there is no synthesis of the factors influencing their mental health. To address this gap, we conducted a scoping review using the socio-ecological model to identify and synthesise these factors.

**Study design:**

A scoping review following JBI methodology and reported according to the Preferred Reporting Items for Systematic Reviews and Meta‐Analyses Extension for Scoping Reviews (PRISMA‐ScR).

**Data sources:**

Medline, Scopus, Embase, CINAHL, PsycINFO, Web of Science, and ERIC were searched for Australian peer-reviewed empirical studies published between January 2015 and April 2026.

**Data synthesis:**

Of the 2,712 publications identified, only 11 met inclusion criteria, highlighting a limited evidence base. Guided by the socio-ecological model, we identified 10 factors influencing mental health. Reported factors at the individual level included functioning in daily living activities, chronic pain, impairment type, and employment status. At the interpersonal level, factors included exposure to bullying and victimisation, sense of belonging, and relationships with teachers and peers. At the organisational level, factors related to education, including the physical design of learning spaces, and the flexibility and accommodations available for navigating the educational environment. No publications addressed community- or policy-level factors. Few included perspectives of young Australians with disability, and none reported co-design. No research was in rural and remote contexts or included Aboriginal and Torres Strait Islander young people.

**Conclusion:**

Evidence on the mental health of young Australians with disability is sparse. This limits the capacity of current responses to address the broader conditions shaping mental health and wellbeing. Action is needed to shift beyond an individual focus toward coordinated, multi-level strategies that address structural drivers of mental health inequities.

**Supplementary Information:**

The online version contains supplementary material available at https://doi.org/10.1186/s12889-026-28583-8.

## Introduction

Good mental health is essential to the overall health and wellbeing of young people as they transition from adolescence into adulthood [1]. This life stage – often referred to as emerging adulthood (typically ages 15 to 24) – is a sensitive developmental period marked by rapid brain development and identity formation [2–4]. As a result, young people are recognised to be at a higher risk of experiencing poorer mental health compared to other age groups. This can profoundly shape their mental health trajectories across their lifespan [1, 3, 5].

Rather than being a fixed or binary state, mental health exists on a continuum and is considered fluid [6, 7]. An individual’s position on this continuum can shift at any time due to a complex interplay of individual, social, and structural influences [3, 6, 8–10]. Among young people, those with disability in Australia face a disproportionately high risk of poor mental health [11–13]. This is due to multiple intersecting factors such as poorer living conditions [14] and higher levels of unemployment, [15] social exclusion, [16] discrimination, [17] violence, and bullying [18]. In Australia, mental health inequities are stark: 32% of young people with disability have poor mental health compared with just 13% of their non-disabled peers [19]. These inequities are also persistent, with longitudinal data showing that young people with disability consistently report poorer mental health over an 18-year period [13].

Despite this elevated risk, we know little about how, when, and where to support young people with disability in improving and maintaining their mental health during this critical transition period [1]. While the influences on mental health are complex, there has been limited synthesis of the factors related to this population’s mental health in order to identify potential points for policy interventions.

Understanding these influences requires attention to context. Marmot’s work on the social determinants of health illustrates that inequities arise from the specific social and structural conditions in which people live [20]. Scholars such as Greenhalgh [21] and Dixon-Woods [22] further demonstrate that evidence and interventions are shaped by the systems, organisational practices, and histories of the settings in which they are embedded. These conditions differ substantially between countries, requiring caution about direct transferability of international findings on mental health to young people with disability in Australia. Australia’s distinctive policy landscape and the fragmented responsibilities across federal and state systems, entrenched rural – urban inequities, its multi-cultural diversity, and the ongoing impacts of colonisation on Aboriginal and Torres Strait Islander communities—creates patterns of risk and access not replicated elsewhere. A national synthesis is therefore essential to identify the influences most relevant to this population and to inform policies and services that are most likely to be effective within Australia’s particular social, cultural and structural environment.

**Fig. 1:**
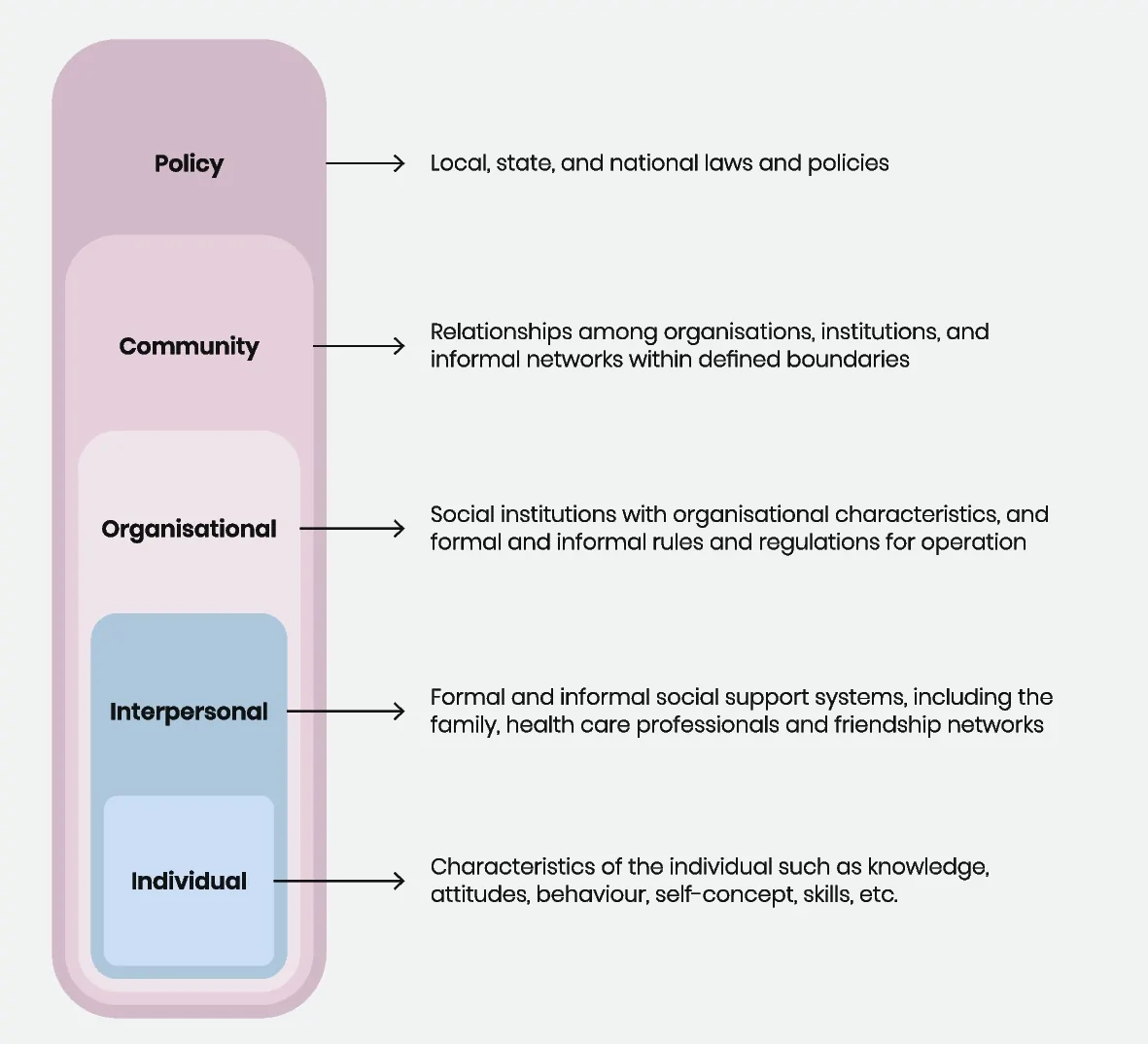
Socio-ecological model proposed by McLeroy and colleagues [26]

The socio-ecological model provides a framework for mapping the complex and interacting factors that influence an individual’s mental health [23–25]. It does this by examining how various layers of the environment – from the intrapersonal to broader societal contexts – shape health behaviour and outcomes, while simultaneously recognising the dynamic, bi-directional relationship that an individual has with their environment. For this review we apply the socio-ecological model proposed by McLeroy and colleagues [26], widely used in public health research [23, 26, 27]. This model conceptualises five nested levels of influence and interaction: individual, interpersonal, organisational, the community, and public policy (Fig. 1). To our knowledge the socio-ecological model has not previously been used to examine the mental health of young people with disability.

This scoping review aims to identify and synthesise the existing literature on the multi-level factors influencing the mental health of young Australians with disability by applying a socio-ecological model. By mapping the factors across these five levels, this review seeks to strengthen understanding about the factors influencing mental health of young Australians with disability and to inform future research, strategies and policy responses.

## Methods

### Study design

We used a scoping review methodology guided by JBI (formerly known as Johanna Briggs Institute), which is well suited to mapping the extent and nature of evidence in response to a broad research question within a specific context [28–31]. The review was conducted in accordance with a published *a priori* protocol (available here:<https://osf.io/u9nps/files/osfstorage>) [32]. Reporting adhered to the Preferred Reporting Items for Systematic Reviews and Meta-Analyses extension for Scoping Reviews (PRISMA-ScR) guidelines [33].

### Research question

The research question was: *What is the nature and extent of research about the factors influencing the mental health of young Australians with disability?*

### Inclusion criteria

Inclusion criteria were established and refined by our study’s review team using the PCC (Population, Concept, Context) framework set out by JBI [31].

#### Population

Our review focused on young people with disability. As there is no universal definition of ‘youth’ or ‘young people’, we adopted the United Nations definition of individuals aged 15 and 24 years [34]. In line with the focus on mental health during youth transitioning to adulthood, we extended the upper range to 29 years, consistent with Arnett’s life course theory of ‘emerging adulthood’ [2]. Publications were excluded if age-specific data could not be disaggregated for this group from the broader sample or if participant age was not reported.

Disability was defined in line with the World Health Organization’s (WHO) International Classification of Functioning, Disability and Health. That is, a long-term health condition or impairment lasting more than six months that results in difficulties – described as activity limitations or participation restrictions – functioning in daily life [35]. Publications that did not address functioning, and or disability were excluded. Consistent with this approach, search terms were limited to *disability* and *impairment* to capture literature engaging with disability as a social and structural experience rather than determined by diagnosis alone.

#### Concepts

We adopted the WHO’s conceptualisation of mental health as *“a state of mental well-being that enables people to cope with the stresses of life, to realize their abilities, to learn well and work well, and contribute to their communities. Mental health is an integral component of health and well-being and is more than the absence of mental disorder.”* (Page 8) [6]

We defined *factors* as conditions, characteristics, or influences that may shape, support, or undermine the mental health of young people. We excluded intervention studies in line with our scope of examining factors influencing mental health.

#### Context

This review only considered publications set in Australia. Multi-country publications were included if data specific to Australia could be identified.

### Types of sources

This scoping review included original research published in the scientific literature. To maintain a clearly defined and manageable scope and support a consistent and reproducible approach to identifying and selecting evidence, grey literature and non-original research publications were excluded. This included reports, study protocols, conference abstracts, conference proceedings, letters to the editor, editorials, commentaries, opinion pieces, books, book chapters, book reviews, and theses.

### Relevant literature search

An initial search of Medline (OVID-SP) and Google Scholar was conducted to identify publications on the topic, and to build a list of relevant search terms with academic librarians and content experts in the field of disability and mental health. Following this initial search, a full search strategy for Medline was developed by an academic librarian (KE) and translated to the following databases: Scopus, Embase, CINAHL, PsycINFO, Web of Science, and ERIC. Database searches were conducted from 1 January 2015 to 7 April 2026. The index year of 2015 was chosen to capture just over a decade of contemporary understandings of the influences on the mental health of young people with disability. The final search strategy for each database can be found in Appendix 1.

### Study selection

Following the search, all identified citations were collated and uploaded into Covidence [36], a web-based review platform, and duplicates removed. Following a pilot test of the eligibility criteria, titles and abstracts were screened first by one reviewer (JB) and then by another (GL, SM, MS, TB, AS, ZA, or SD). Full texts were independently screened by two of three reviewers (JB, GL, ZA). Any disagreements that arose between the reviewers at each stage of the selection process were resolved by the lead author (JB) or after discussion with other reviewers.

### Data extraction

A data extraction template was developed *a priori* in Covidence and piloted on four randomly selected publications and updated accordingly. The template included key characteristics of each publication, such as study setting, study population, methods, and the factors influencing mental health. Data extraction was undertaken by one reviewer (JB) and 100% cross-checked by two others (ZA and GL). Any uncertainties during the data extraction process were resolved through discussion.

### Data analysis and presentation

Extracted data were exported from Covidence to Excel for analysis. Summary statistics describing the study’s characteristics – such as the design, methods, and setting – were tabulated. An inductive qualitative content analysis was undertaken on the extracted data to identify factors influencing mental health, following the steps outlined by Elo and Kyngäs [37]. Factors were then coded to the socio-ecological model using McLeroy et al.’s five levels of influence. This process was undertaken by JB and cross checked by ZA. This recursive process involved iterative reading, coding, and categorisation of data. To ensure consistency and conceptual clarity, emerging factors and their corresponding socio-ecological levels were discussed, cross-checked, and refined through regular discussions and review cycles.

## Results

### Search results and study selection

**Fig. 2:**
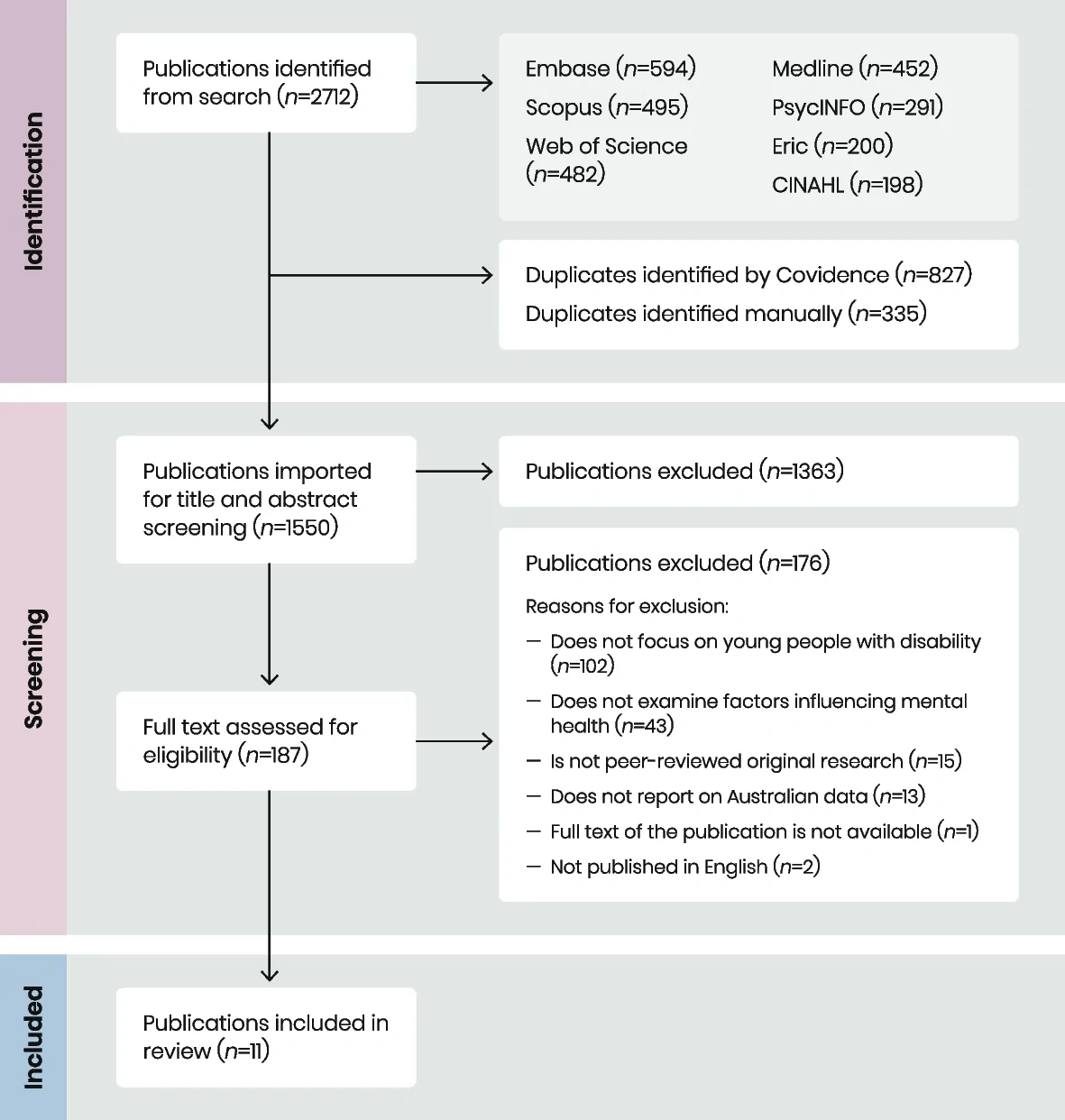
Preferred reporting of items for systematic reviews and meta-analysis extension for scoping reviews (PRISMA-ScR) flow diagram

The search identified 2,712 publications. After duplicate removal, title, and abstract screening, and full text review, 11 publications were included (Fig. 2).

### Characteristics of included publications

The characteristics of the eleven included publications are summarised in Table 1, with more detailed information on the characteristics of included publications provided in Appendix 2. Four publications reported on Australia-wide data. Six of the eleven did not report any rurality settings for participants; four were set in urban locations; and one reported data from participants across mixed rurality settings (metropolitan, inner regional, and outer regional/remote) [15]. No publications examined the factors influencing mental health among Aboriginal and Torres Strait Islander young people with disability, and none explored multicultural diversity.

**Table 1:** Summary of included publications

Category	Sub-category	Number of publications and references
Geographic spread	Australia-wide	4 [15, 18, 38, 39]
	WA	2 [40, 41]
	QLD	1 [42]
	VIC	1 [43]
	WA, VIC, NSW	1 [44]
	NSW and ACT	1 [45]
	VIC and NSW	1 [46]
Rurality of participants	Not stated	6 [18, 38–40, 44, 45]
	Urban	4 [41–43, 46]
	Mixed rurality for study participants	1 [15]
Study design	Quantitative	7 [15, 18, 38–40, 43, 44]
	Qualitative	4 [41, 42, 45, 46]
Participant group	Carers and family members as proxies	2 [40, 44]
	Self-report and proxy	3 [18, 41, 43]
	Self-report (young person with disability)	6 [15, 38, 39, 42, 45, 46]
Impairment type*	Impairment (type not specified)	3 [15, 18, 39]
	Intellectual disability	3 [38, 41, 44]
	Down syndrome	2 [40, 44]
	Cerebral palsy	2 [43, 45]
	Autism spectrum disorder	2 [42, 46]
Population level data sets used*	Household, Income and Labour Dynamics in Australia Survey	2 [15, 39]
	Longitudinal Study of Australian Children	1 [18]
	Needs Opinions and Wishes database (WA-based)	2 [40, 44]
	Australian Child to Adult Development survey (NSW and VIC-based)	1 [44]
	Intellectual Disability Exploring Answers (WA-based)	1 [40]
Mental health outcomes*	General mental health	7 [15, 18, 39, 41, 42, 45, 46]
	Depressive symptoms	3 [38, 40, 44]
	Anxiety	2 [40, 44]
	Psychological wellbeing	1 [43]
Mental health tools used	Mental Health Inventory (MHI-5) subscale from the Short Form-36 (SF-36) general health scale	2 [15, 39]
	Developmental Behaviour Checklist – Child (96 items) and Adult (107 items)	2 [40, 44]
	Quality of Life Instrument for Young Adults (YAQOL)	1 [43]
	Strengths and Difficulties Questionnaire (SDQ)	1 [18]
	Centre for Epidemiologic Studies Depression scale adapted for adolescents with an Intellectual Disability (CES-D-ID)	1 [38]

^*^Numbers may not add up as publications might have examined more than one mental health outcome, impairment type or used numerous databases in one publication

*ACT* Australian Capital Territory, *NSW* New South Wales, *QLD* Queensland, *VIC* Victoria, *WA* Western Australia

### Study methods and data sources

Most of the publications were quantitative in design (*n* = 7) [15, 18, 38–40, 43, 44], the remaining were qualitative (*n* = 4) [41, 42, 45, 46]. Of the 11 publications, six elicited responses directly from young people with disability, two relied on families or carers as proxies, and three used a mix of self- and proxy-reporting.

Of the seven quantitative publications, five drew on pre-existing data sources, comprising population-level datasets from five distinct surveys. Three publications used two different national datasets [15, 18, 39], and two publications used three different state-based population-level datasets [40, 44].

Primary data collection methods – semi-structured interviews [41, 42, 45, 46] and cross-sectional surveys [38, 43] – were employed in six of the 11 publications. One qualitative study that used semi-structured interviews augmented with photo elicitation methods and emotion cards [46].

### Impairment type

Eight publications focused on specific impairment or diagnostic groups, including intellectual disability [38, 41, 44], Down syndrome [40, 44], autism spectrum disorder [42, 46], and cerebral palsy [43, 45]. Three publications did not identify a specific impairment [15, 18, 39].

### Mental health descriptors

Mental health descriptors included: general mental health [15, 18, 39, 41, 42, 45, 46]; depressive symptoms [38, 40, 44]; anxiety [40, 44]; and psychological wellbeing [43]. There were five different mental health measurement tools used in the seven quantitative publications (Table 1), reflecting methodological heterogeneity in the constructs assessed.

### Factors influencing the mental health of young people with disability

Overall findings from the extracted publications are described under each of the five levels of the socio-ecological model and summarised in Table 2.

**Table 2:** Factors influencing mental health of young people with disability in Australia by socio-ecological model levels

Socio-ecological level	Factors
Individual (*n* = 6) [15, 39, 40, 43–45]	Functioning in activities of daily living [40, 43]
	Employment status [15, 39]
	Chronic pain [45]
	Impairment type [44]
Interpersonal (*n* = 4) [18, 38, 41, 42]	Bully-victimisation [18, 38]
	Sense of belonging [41]
	Relationships with teachers [38, 42]
	Relationships with peers [42]
Organisational (*n* = 2) [42, 46]	Physical environment and design of university spaces [46]
	Curriculum and school environment [42]
Community	None reported
Policy	None reported

*n* number of publications

#### Individual level

Six publications identified four individual level factors that influenced mental health [15, 39, 40, 43–45]. Functional ability [40, 43] and chronic pain [45] were associated with poorer mental health, while employment (both part- and full-time) was protective, [15, 39] explaining about 20% of the relationship between disability and mental health [15]. One study found that individuals with Down syndrome experienced fewer depressive and anxiety symptoms than those with other causes of intellectual disability [44].

#### Interpersonal level

Four studies identified interpersonal level factors shaping mental health [18, 38, 41, 42]. Teacher-student relationships were particularly important – conflict or negative interactions increased stress and depressive symptoms [38, 42]. Bullying and victimisation were linked to poorer mental health [18], while positive peer relationships and a sense of belonging promoted better mental health [41, 42].

#### Organisational level

Two publications identified organisational level factors that influenced mental health for autistic students [42, 46]. In schools, noisiness, crowding, heavy workloads, handwriting demands, and inflexible work organisation were associated with stress and anxiety [42]. In universities, supportive environments – such as access to calming outdoor areas—reduced anxiety, whereas poor signage and unclear layouts had negative effects on students’ mental health [46].

#### Community and policy level

No publications identified community or policy level factors.

## Discussion

This scoping review contributes to the evidence base by identifying factors reported to influence the mental health of young people with disability. Guided by the socio-ecological model, we identified 10 factors across three of the five levels of this model that influenced poorer or better mental health. Reported factors at the individual level included functioning in daily living activities, living with chronic pain, impairment type, and employment status. At the interpersonal level, factors included exposure to bullying and victimisation, having a sense of belonging, and developing relationships with teachers and peers. At the organisational level, factors related to education, including the physical design of learning spaces, and the flexibility and accommodations available for navigating the educational environment. No publications addressed community- or policy-level factors influencing mental health.

A key finding of this review is the lack of peer-reviewed literature with Australian data about factors influencing the mental health of young people with disability. This gap is worrying given their consistently poorer mental health outcomes compared with their non-disabled peers [13]. A second key finding is that no publication identified community- or policy level factors. This pattern reflects a broader trend in which, despite the long-standing use of socio-ecological models and systems-thinking approaches, research on mental health predominantly concentrates on proximal factors [47–49]. Future research should prioritise interactions across all levels and also, their cumulative effects on mental health. Young people with disability often fall between the gaps – neither included in policies designed for other young people nor adequately considered in terms of how factors influencing their mental health may differ from their peers. Addressing this will enable a more nuanced understanding of the influences on the mental health of young people with disability and support the development of appropriately targeted interventions and policies.

At the individual level, several publications identified chronic pain, impairment type and reduced capacity to perform daily activities as factors associated with mental health. This is not to suggest that these are predictive or determining factors in an individual’s mental health. Rather, as reflected in the International Classification of Functioning, Disability and Health [35] restrictions to everyday living and challenges to participation in society arise from interaction between health conditions or impairments and the social and structural environments in which people live [35].

Interpersonal and organisational factors appeared less frequently but remain critical to understanding mental health for young people with disability. For example, only one Australian study addressed bullying and victimisation despite national data showing that 75% of students with disability experienced bullying and 72% were excluded from school activities or events in 2024 [50]. Organisational factors – particularly within schools and universities – were linked to environmental stressors such as noise and rigidity. These findings demonstrate that organisational contexts, particularly educational settings, can play a significant role in shaping the mental health of young Australians with disability during emerging adulthood.

There were significant gaps in research on rural and remote contexts, including no studies examining mental health of Aboriginal and Torres Strait Islander young people with disability. These omissions are concerning given the higher prevalence of disability in Aboriginal and Torres Strait Islander communities and those living in rural areas [51], as well as the distinct social and environmental stressors experienced by young people outside metropolitan centres [52].

The most striking gap in the reviewed literature was the absence of voice of young people with disability with no study incorporating co-design elements. Several studies also relied partly or entirely on proxy reporting from parents or carers. While at times necessary, proxy reporting raises questions about whose perspective on mental health is captured. Together, the absence of co-design and reliance on proxy reporting mean that the perspectives of young people with disability are less likely to inform policy and practice initiatives that better centre their voices and lived experiences [53, 54].

### Strengths and limitations

Strengths of the review are: 1) a published *a priori* protocol, which enhanced transparency and reproducibility; 2) the application of a well-established framework, the socio-ecological model; 3) the rigour of two reviewers independently conducting title and abstract screening, full text review and data extraction, and engaging in robust discussions to reach consensus at every stage; and 4) a search strategy designed with an experienced academic librarian.

There were several limitations. Relevant evidence may have been missed due to the exclusion of grey literature, including government and non-government reports. In addition, publications may not have been identified in the search if they did not use the terms ‘disability’ or ‘impairment’, including studies on specific diagnoses. However, this approach reflected our intention to capture literature concerned with the disabling social and structural dimensions of experience, rather than health conditions alone.

## Conclusions

Overall, we found a remarkable scarcity of evidence, with most research focused on individual level factors, a small number addressing interpersonal or organisational influences, and none exploring community- or policy-level factors. Particular attention is needed to rural and remote contexts, including Aboriginal and Torres Strait Islander young people with disability. Building a stronger evidence base is essential to inform appropriate policy, service design, and interventions that address the social determinants of mental health and that are meaningful to young Australians with disability as they transition to adulthood.

## Supplementary Information

Supplementary Material 1.

## Data Availability

All data and materials generated or analysed are included in this article.

## Abbreviations

ACT: Australian Capital Territory
NSW: New South Wales
PRISMA-ScR: Preferred Reporting Items for Systematic Reviews and Meta-Analyses extension for Scoping Reviews
QLD: Queensland
VIC: Victoria
WA: Western Australia
WHO: World Health Organization
