# Supplementary material for: Factors influencing the mental health of young people with disability in Australia: a systematic scoping review
Source: BMC Public Health. 2026 Jul 22;26:2613. doi: 10.1186/s12889-026-28583-8 (PMC13584278; doi:10.1186/s12889-026-28583-8)
Supplement: Supplementary file 1 [file 12889_2026_28583_MOESM1_ESM.docx]

**Appendix 1**

**Ovid MEDLINE(R)**

1	exp Persons with Disabilities

2	(disab* or handicap*).mp.

3	1 or 2

4	Adolescent/ or Young Adult/ or exp Students/ or exp Minors/

5	(adolescen* or "young adult*" or young people* or young person* or "youth*" or "teen*" or "high school*" or "secondary school*" or "college student*" or "university student*" or "emerging adult*" or "early adulthood" or "juvenile" or "between 15 to 30" or "between 15-30").mp.

6	4 or 5

7	mental health/ or Anxiety/ or Depression/ or Stress, Psychological/

8	((wellbeing or well-being or welbeing or wellness or empowerment* or flourishing) and (psychological* or mental* or emotional*)).mp.

9	("mental health*" or Anxi* or Depress* or "Psychological stress*").mp.

10	8 or 9

11	exp Australia/

12	(Australia or New South Wales or Queensland or Victoria or Australian Capital Territory or South Australia or Western Australia or Northern Territory or Tasmania).mp.

13	(NSW or NT or VIC or QLD or ACT or WA or NT or SA).tw.

14	11 or 12 or 13

15	3 and 6 and 10 and

16	limit 15 to yr="2000 -Current"

**Embase Classic+Embase**

1	disabled person/

2	(disab* or handicap*).mp.

3	1 or 2

4	adolescent/ or young adult/ or student/ or "minor (person)"/ or juvenile/ or high school student/ or college student/ or university student/

5	(adolescen* or "young adult*" or young people* or young person* or "youth*" or "teen*" or "high school*" or "secondary school*" or "college student*" or "university student*" or "emerging adult*" or "early adulthood" or "juvenile" or "between 15 to 30" or "between 15-30").mp.

6	4 or 5

7	mental health/ or Anxiety/ or depression/ or mental stress/

8	((wellbeing or well-being or welbeing or wellness or empowerment* or flourishing) and (psychological* or mental* or emotional*)).mp.

9	("mental health*" or Anxi* or Depress* or "Psychological stress*" or "mental stress").mp.

10	7 or 8 or 9

11	exp Australia/

12	(Australia or New South Wales or Queensland or Victoria or Australian Capital Territory or South Australia or Western Australia or Northern Territory or Tasmania).mp.

13	(NSW or NT or VIC or QLD or ACT or WA or NT or SA).tw.

14	11 or 12 or 13	91

15	3 and 6 and 10 and 14

16	limit 15 to yr="2000 -Current"

**APA PsycInfo**

1	exp disabilities/

2	(disab* or handicap*).mp.

3	1 or 2

4	exp Students/

5	(adolescen* or "young adult*" or young people* or young person* or "youth*" or "teen*" or "high school*" or "secondary school*" or "college student*" or "university student*" or "emerging adult*" or "early adulthood" or "juvenile" or "between 15 to 30" or "between 15-30").mp.

6	4 or 5

7	mental health/ or Anxiety/ or Psychological Stress/

8	((wellbeing or well-being or welbeing or wellness or empowerment* or flourishing) and (psychological* or mental* or emotional*)).mp.

9	("mental health*" or Anxi* or Depress* or "Psychological stress*").mp.

10	7 or 8 or 9

11	(Australia or New South Wales or Queensland or Victoria or Australian Capital Territory or South Australia or Western Australia or Northern Territory or Tasmania).mp.

12	(NSW or NT or VIC or QLD or ACT or WA or NT or SA).tw.

13	11 or 12

14	3 and 6 and 10 and 13

15	limit 14 to yr="2000 -Current"

**Scopus**

( TITLE-ABS-KEY ( disab* OR handicap* ) AND TITLE-ABS-KEY ( adolescen* OR "young adult*" OR "young people*" OR "young person*" OR youth* OR teen* OR "high school*" OR "secondary school*" OR "college student*" OR "university student*" OR "emerging adult*" OR "early adulthood" OR juvenile OR "between 15 to 30" OR "between 15-30" ) AND TITLE-ABS-KEY ( ( ( wellbeing OR well-being OR welbeing OR wellness OR empowerment* OR flourishing ) AND ( psychological* OR mental* OR emotional* ) ) ) OR TITLE-ABS-KEY ( "mental health*" OR anxi* OR depress* OR "Psychological stress*" ) AND TITLE-ABS-KEY ( australia OR "New South Wales" OR queensland OR victoria OR "Australian Capital Territory" OR "South Australia" OR "Western Australia" OR "Northern Territory" OR tasmania ) ) AND PUBYEAR > 1999 AND PUBYEAR < 2026

**Web of Science**

1 disab* OR handicap* (Topic) and Preprint Citation Index (Exclude – Database)
2 adolescen* OR "young adult*" OR "young people*" OR "young person*" OR youth* OR teen* OR "high school*" OR "secondary school*" OR "college student*" OR "university student*" OR "emerging adult*" OR "early adulthood" OR juvenile OR "between 15 to 30" OR "between 15-30" (Topic) and Preprint Citation Index (Exclude – Database)
3 ( ( wellbeing OR well-being OR welbeing OR wellness OR empowerment* OR flourishing ) AND ( psychological* OR mental* OR emotional* ) ) (Topic) and Preprint Citation Index (Exclude – Database)
4 "mental health*" OR anxi* OR depress* OR "Psychological stress*" (Topic) and Preprint Citation Index (Exclude – Database)
5 australia OR "New South Wales" OR queensland OR victoria OR "Australian Capital Territory" OR "South Australia" OR "Western Australia" OR "Northern Territory" OR tasmania (Topic) and Preprint Citation Index (Exclude – Database)
6 #3 OR #4 and Preprint Citation Index (Exclude – Database)
7 #1 AND #2 AND #5 AND #6 and Preprint Citation Index (Exclude – Database)
8 #1 AND #2 AND #5 AND #6 and Preprint Citation Index (Exclude – Database) and 2000 or 2001 or 2002 or 2003 or 2004 or 2005 or 2006 or 2007 or 2008 or 2009 or 2010 or 2011 or 2012 or 2013 or 2014 or 2015 or 2016 or 2017 or 2018 or 2019 or 2020 or 2021 or 2022 or 2023 or 2024 or 2025 (Publication Years)

**Cinahl**

S1 (MH "Persons with Disabilities+") OR (disab* OR handicap*)

S2 (MH "Adolescence " OR MH "Young Adult" OR MH "Students " OR MH "Students with Disabilities" OR MH "Minors (Legal)") OR (adolescen* OR "young adult*" OR youth* OR teen* OR "high school*" OR "secondary school*" OR "college student*" OR "university student*" OR "emerging adult*" OR "early adulthood" OR juvenile OR "between 15 to 30" OR "between 15-30")

S3 MH "Anxiety+" OR MH "Depression+" OR MH "Stress, Psychological+" OR ("mental health*" OR Anxi* OR Depress* OR "Psychological stress*" )

S4 ((wellbeing OR well-being OR welbeing OR wellness OR empowerment* OR flourishing ) AND (psychological* OR mental* OR emotional* ))

S5 S3 OR S4

S6 “Australia+" OR MH "Western Australia" OR MH "South Australia" OR MH "Victoria" OR MH "Tasmania" OR MH "Queensland" OR MH "Northern Territory" OR MH "New South Wales" OR MH "Australian Capital Territory" OR australia OR "New South Wales" OR queensland OR victoria OR "Australian Capital Territory" OR "South Australia" OR "Western Australia" OR "Northern Territory" OR tasmania

S7 S1 AND S2 AND S5 AND S6

S8 S1 AND S2 AND S5 AND S6

**ERIC (Education)**

1	exp Disabilities/

2	(disab* or handicap*).mp.

3	1 or 2

4	exp adolescents/ or exp Students/ or exp Students with Disabilities/ or exp high schools/ or exp College Students/ or exp Young Adults/ or exp College Students/

5	(adolescen* or "young adult*" or young people* or young person* or "youth*" or "teen*" or "high school*" or "secondary school*" or "college student*" or "university student*" or "emerging adult*" or "early adulthood" or "juvenile" or "between 15 to 30" or "between 15-30").mp.

6	4 or 5

7	exp Mental Health/ or anxiety/ or anxiety disorders/ or exp "depression (psychology)"/

8	((wellbeing or well-being or welbeing or wellness or empowerment* or flourishing) and (psychological* or mental* or emotional*)).mp.

9	("mental health*" or Anxi* or Depress* or "Psychological stress*").mp.

10	7 or 8 or 9

11	(Australia or New South Wales or Queensland or Victoria or Australian Capital Territory or South Australia or Western Australia or Northern Territory or Tasmania).mp.

12	(NSW or NT or VIC or QLD or ACT or WA or NT or SA).tw.

13	11 or 12

14	3 and 6 and 10 and 13

15	limit 14 to yr="2000 -Current"

**Appendix 2: Summary of included publications, by alphabetical order of first author**

| **Authors \| Location** | **Stated aims or objectives** | **Impairment type** | **Design \|data collection and analysis** | **Population characteristics** | **Relevant exposure and outcome measurements / tools** | **Findings relevant to the aims of the review** | **Factors identified** |
| --- | --- | --- | --- | --- | --- | --- | --- |
| Foley K, Bourke J, et al (2015)(1)  WA  Rurality: not stated | “The aim of this study was to describe the trajectories of sub-scales of behaviour, including depressive symptoms, communication disturbance, anxiety, disruptiveness, and social relating abilities, for young adults with Down Syndrome.” (Abstract, Pg. 1) | Down Syndrome | **Design**: Longitudinal cohort study using a population-based sampling frame  **Data source:** A sample of participants identified through the *Needs Opinions and Wishes (NOW)* database and the *Intellectual Disability Exploring Answers (IDEA)* database (covers ~97% of Down syndrome cases in WA).  **Recruitment:** Families invited by mail (direct or via Disability Services Commission). Data collected via paper questionnaires, online, or telephone interviews.  **Timeframe:** Three waves – Wave 1 (2004), Wave 2 (2009), Wave 3 (2011)  **Analysis:** Regression models with random intercepts and generalised estimating equations. | Wave 1: n = 255 (mean age 13.9 yrs; range 3–24)  Wave 2: n = 191 (mean 21.8 yrs; range 15–29)  Wave 3: n = 188 (mean 23.5 yrs; range 16–31) | ***Exposure:***  **Functioning,**  - Wave 1: Functional Independence Measure for Children (WeeFIM)  - Waves 2 & 3: Index of Social Competence (ISC)  ***Mental Health Outcome:***  Developmental Behaviour Checklist – Child (96 items) and Adult (107 items), with 6 subscales, two subscales were related to mental health. | Depressive symptoms persisted over time increasing with age, with small decline from wave 1 to 3 (coeff – 0.003, 95% CI -0.007, -0.0001).  “Those who were  reported as having lower functioning were reported as exhibiting  significantly more problem behaviours across every subscale …” (pg. 4) including the depressive symptoms and anxiety subscales.  Figure 4, Pg 5 (Note: No 95% confidence intervals were presented) | Lower functioning in activities of daily life *(Individual)* |
| Foley K, Taffe J, et al. 2016 (2)  WA, VIC, NSW  Rurality: not stated | “ … to compare changes in emotional and behavioural problems for young people with intellectual disability with and without Down Syndrome as they transition into adulthood in two different Australian cohorts” (Abstract, Pg. 1) | Down Syndrome compared to people with Intellectual Disability of another cause | **Design**: Longitudinal cohort analysis  **Data source:** using pooled data from two population-based databases: a) *Needs Opinions and Wishes (NOW)* database (WA) b) *Australian Child to Adult Development (ACAD)* survey (NSW and VIC).  Adults with Down syndrome (from WA, NSW, VIC) were compared to adults with other causes of intellectual disability (from NSW and VIC).  **Recruitment:** For ACAD, families sampled from health, education and family agencies across six census districts across NSW and Vic. Not reported for NOW. Questionnaires administered to families via paper, over the internet or via telephone surveys  **Timeframes:** Data time points from ACAD Time point 1 (1991–1992), time point 3 (1999) and time point 4 (2002–2003) were combined with the data from the NOW database time point 2 (2004), time point 3 (2009) and timepoint 4 (2011)  **Data analysis:** Random effects regression. | **Sample size and age:**  Wave 1: Down syndrome (n = 323, M = 13.42, SD = 5.70); other intellectual disability (n = 466, M = 11.78, SD = 4.02) Wave 2: Down syndrome (n = 202, M = 20.96, SD = 4.57); other intellectual disability (n = 340, M = 19.20, SD = 4.16) Wave 3: Down syndrome (n = 200, M = 23.54, SD = 4.44); other intellectual disability (n = 335, M = 23.23, SD = 4.19) | ***Mental Health Outcome:***  Developmental Behaviour Checklist (DBC)–Child (96 items) & Adult (107 items); parent/carer report; with 6 subscales: two subscales were related to mental health. | Individuals with Down syndrome had fewer depressive (estimated mean difference = –0.122, SE = 0.38, *p* < .01) and anxiety symptoms (estimated mean difference = –0.069, SE = 0.21, *p* < .01) than those with other causes of intellectual disability across all waves. | ~~I~~mpairment type (*Individual)* |
| Jiang B, et al. 2016 (3)  Royal Children’s Hospital, Melbourne, VIC  Rurality: Urban | “This cross-sectional analysis compares the quality of life (QoL) of a cohort of young Australian adults with cerebral palsy with a cohort of able-bodied peers to explore the relationship between QoL and impairments, functioning and social participation.: (Abstract, Pg. 673) | Cerebral Palsy | **Design:** Cross-sectional survey, comparative study.    **Recruitment:** Young adults born between 1976 and 1985 were identified from the Victorian Cerebral Palsy Register.  Survey mailed and either self-administered or by proxy.  **Analysis**: Multivariate linear regression models. | **Sample size:** 335 young people with cerebral palsy or their proxy self-reported their quality of life. Self-report group (n=207); proxy-report group (n=128).  **Age group:** Mean age was 24.7yrs (s.d = 2.8) | ***Exposure:***  **Independence in self-care activities**, using Barthel Index which has 10 domains comprising feeding, bathing,  grooming, dressing, bladder management, bowel management,  toilet use, transfers, mobility, and ability to use stairs.  ***Mental Health Outcome:* Quality of Life** Instrument for Young Adults (YAQOL) across five domains: one domain being psychological wellbeing. | Functional independence was positively associated with psychosocial wellbeing (β (standard coefficients = 0.23, p=0.003).  Impairment type, employment status, living situation, marital status, educational achievement, income, location of residence and unmet healthcare needs were not associated  with psychological well-being in the multivariate regression analysis. | Functional independence *(Individual)* |
| King T, et al. 2018 (4)  Australia wide  Rurality: not stated | “We applied a casual mediation approach to examine the extent to which bullying-victimisation mediates the relationship between disability and mental health …” (Pg. 1404) | Disability as a composite group compared to no disability.  To minimise potential conflation between exposure (disability) and outcome (mental health) authors used the LSAC measure of disability that did not include mental illness. | **Design & Source:** Secondary analysis of Longitudinal Study of Australian Children (LSAC), comparative study.  **Study dates**: Cohort K was used, 2010–2014, Waves 4–6.  **Analysis:** Parametric regression. | **Sample size**: *n =* 2728 no disability and *n* = 109 with disability  **Age group:** 14-18yrs, reporting on mental health and bullying was at 14–15 years. | ***Exposure:*** **Bully – victimisation,** adolescents and the primary household informant were asked 11 questions about bullying – victimisation. In analysis used adolescence, teacher and parent-reported bullying – victimisation at age 14–15yrs.  ***Mental Health Outcome:*** Measured with the Strengths and Difficulties Questionnaire (SDQ). | Among those with disability bullying-victimisation was consistently associated with worse mental health.  “There was strong evidence of mediation by bullying-victimisation for the relationship between disability and MH (Natural Indirect Effect of 1.01, 95% CI: 0.30– 1.80), indicating that almost half (46%, 95% CI: 15–117%) of the excess in adolescent-reported ‘Total Difficulties’ due to disability was mediated through bullying-victimization.” (Pg. 1407)  This finding was consistent across all combinations of informants for mental health and bullying, except for the combination of adolescent-reported bullying and teacher-reported mental health which found no evidence of a natural indirect effect. | Bullying – victimisation *(Interpersonal)* |
| Milbourn B, et al. 2020 (5)  WA  Rurality: Urban | “This study analysed experiences and possible benefits to occupational wellbeing of young men with intellectual disability in an intergenerational mentoring program based on Australian Men’s Sheds …” (Abstract, Pg. 121) | Mild- moderate intellectual disability | **Design:** Qualitative, semi-structured interviews at end of intergenerational mentoring program to explore occupational wellbeing experiences, 17–24 yrs. The Men’s Shed program took place at seven different metropolitan community-based Men's Sheds.  **Recruitment:** Participants recruited rom graduating mentees of the Men’s Shed program and their family members.  **Analysis:** Deductive content analysis according to the five occupational wellbeing domain descriptors. | **Sample size:** *n* = 13 (8 individual mentees and 5 parents of mentees)  **Age group:** Mentees were all aged 17–24 yrs. | Qualitative responses | “Parents of the mentees identified how being part of a group where the primary focus was not therapy made a big difference to the mentees wellbeing.” (Pg. 127)  “Families alluded to the importance of camaraderie, which created a feeling of belonging.” (Pg. 127) | Sense of belonging *(Interpersonal)* |
| O’ Connor M, et al. 2024 (6)  VIC and NSW  Rurality: Urban | “This study aimed to capture and understand autistic student experiences in navigating and participating in the physical spaces in an Australian university.” (Pg. 38) | Autism spectrum disorder | **Design:** Qualitative phenomenological design. Semi-structured interviews, photo elicitation, and emotion cards.  **Recruitment:** Disseminated study information via the university’s Disability Support Services, the Autism@Uni website and portal, advertisements on electronic billboards and printed flyers on campus, and by directly emailing participants already involved in mentoring programs for autistic students at the university.  **Analysis:** Reflexive thematic analysis | **Sample size & age:** Seven autistic university students aged 19–37 years (mean 24 years; standard  deviation = 5.84) participated in the study. | Qualitative measures  “We also utilized a set of emotion cards in the interviews.  Participants were asked to select three cards or self-identify  three emotions that best described their feelings within  each space. These cards did not form part of the results of the  study but were used to support the conversation …” (Pg. 39) | “Participants described access to outdoor spaces on campus  as an enabler, with Blake explaining how access to fresh air  had a ‘‘little bit of a calming effect.’’ (Pg. 41)  “Several participants raised navigational issues as a source  of anxiety, with comments referring to feeling ‘‘lost,’’ disorientated or anxious. Participants described the lack of signage and information about some spaces on the university website  as something the university needed to address, with Jordan  reporting that this would reduce anxiety about unfamiliar  spaces.” (Pg. 42)  “… comfort within a space was also closely linked with their mood and, therefore, their level of engagement with their studies and peers. For example, Alex noted that spaces could be ‘‘powerful enough to change [their] mood,’’  with some environments making them anxious or agitated. Several participants also reflected on how higher levels of comfort at university improved their mood, as they were less anxious, less fatigued, and calmer. This had a flow-on  effect beyond their university studies to their social interactions and long-term functioning. Participants described how uncomfortable spaces made it more challenging  to enjoy time spent with others, thus decreasing social opportunities.” (Pg.43)  “Some participants said that they attended more often once they identified comfortable university spaces, as familiarity reduced their anxiety.” (Pg. 43) | Physical environment & design of university spaces *(Organisational)* |
| Olivier E., et al. 2020 (7)  Australia and Canada  Rurality: not stated | “This study assesses the moderating role of two facets of teacher-student relationships – warmth and conflict – on the association between peer victimisation and depressive symptoms.” (Abstract, Pg. 1) | Intellectual disability (mild and moderate) | **Design:** Cross sectional survey. Survey completed by students in small groups using a read-aloud assisted procedure.  **Recruitment** from Australian (*n* = 253) and Canadian (*n* = 142) secondary schools.  **Analysis**: Hierarchical multiple regressions.  ‘A final verification was made to see whether any of these associations differed as a function of youth country or origin. This was not found to be the case.’ (Pg. 7) | *n* = 395 students, aged 11–22 years with mild (*n* = 174) and moderate (*n* = 180) intellectual disability. 86.2% of total sample attended a regular school and 13.8% attended a special school for students with disabilities. | ***Exposure:***  **Peer-victimisation**, The victimisation scale includes 17 items covering physical, verbal, and relational victimisation.  **Teacher-student relationships,** Short form of the Teacher-Student Relationship Scale focuses on students  perception of the quality of their relationship with their teachers in terms of warmth and conflict.  *Mental Health Outcome:*  **Depressive symptoms,** Center for Epidemiologic Studies Depression Scale adapted for adolescents  with an Intellectual Disability (CES-D-ID) | Peer- victimisation associated with depressive symptoms (b = .116 (0.17) ** β .313) (Table 2, Pg 6)  Teacher - Student Relationship (Warmth) not associated with depressive symptoms (b =.-.005 (0.31) β -.011) (Table 2, Pg 6)  Teacher – Student Relationship (Conflict) associated with depressive symptoms (b =.180 (.029) ** β -.390) (Table 2, Pg 6) | Victimisation by peers  *(Interpersonal)*  Teacher-Student relationship conflict  *(Interpersonal)* |
| Ostojic et al. 2022(8)  NSW and ACT  Rurality: not stated | “To explore the lived experiences of pain in children and young people with cerebral palsy.” (Abstract, Pg. 364) | Cerebral Palsy | **Design**: Qualitative study using face-to-face semi-structured interviews.  **Recruitment:** Participants were recruited through the rehabilitation department of the Sydney Children’s Hospital Network and the NSW/ACT cerebral palsy registers (state/territory population-based registers).  **Analysis:** Data were analysed using interpretative phenomenological analysis. | **Sample size & age:** 10 participants (mean age 14 yrs 5 months, SD 2 yrs). | Qualitative measures | “Ivan spoke of the toll that chronic pain had on their mental health and subsequent participation: ‘because of my physical knew thing it starts to get me in a mental loop so I quite often miss out on things … sometimes because of how bad it is, I just kind of don’t feel like doing anything.’ Ivan recounted one period where chronic pain had a profound impact on their mental health and social life..” (Pg. 368)  “For Beth, the belief that ‘I’ll be in pain the rest of my life’ became overwhelming at times, describing feeling like an ‘emotional wreck’. (Pg. 368) | Chronic pain *(individual level)* |
| Saggers B. 2015(9)  Brisbane, QLD.  Rurality: Urban | “The aim of this article is to identify aspects of schooling that were considered positive and enabling, as well as to pinpoint common inhibitors that may prevent students with autism spectrum disorder taking full advantage of their educational experiences ...” (Pg. 2) | Autism spectrum disorder | **Design:** Qualitative data were collected through two face-to-face semi-structured interviews.  **Recruitment:** Criterion-based purposive sampling of participants from a large mainstream public government high school in Brisbane (average enrolment ~1,300 students), which provides support to approximately 80 students with disability.  **Analysis**: Data were analysed using constant comparison methods and compared with themes identified in a previous study. | **Sample size**: *n* = 9  **Age group:** Not stated, though authors note participants enrolled in high grades 8–12. | Qualitative measures | “A significant number of the students interviewed in this study found classroom noise, teachers yelling and crowds in the school environment something which could increase their stress levels and make it difficult for them to cope.” (Pg. 6)  “Additional stressors identified in this study were curriculum-related issues, including workload, homework and handwriting. Specifically, students identified tight work schedules with too much summative assessment due all at once, activities requiring a lot of handwriting, heavy workloads  created by assignments, the amount of non-preferred work they were required to do and getting things handed in on time as central to their stress and anxiety.” (Pg. 6)  “These findings  suggest that to assist students to reduce levels of stress and anxiety, further consideration needs to be given to how workload is presented, organised, and negotiated with students on the spectrum.” (Pg. 6)  “It was strongly evident from the discussions with students with an ASD that they felt a constant need to negotiate difference within the mainstream setting. This was reflected in their relationships with peers, the anxiety and stress they felt in negotiating school life and the issues they had in relation to working with teachers.” (Pg. 7) | Curriculum & school environment  *(Organisational)*  Peer relationships  *(Interpersonal)*  Relationships with teachers  *(Interpersonal)* |
| Shields M, et al. 2023(10)  Australia -wide  Rurality: Mixed (Metro, inner regional, outer regional/remote) | “We aimed to assess if the disability to mental health association was mediated by employment status amount young adults aged 20–35 years.” (Abstract, Pg. 498) | Disability as a composite | **Design:** Longitudinal cohort study  **Data source:** *Household, Income and Labour Dynamics in Australia (HILDA) Survey*  **Timeframe:** 4 consecutive years (2016–2019)  **Analysis:** Casual mediation analysis | **Sample size and age:**  n = 3,435 participants (3,058 without disability and 377 with disability). Mean age of participants with disability: 27.6 years (SD = 4.3). Participants ruralitty 65% metro; 25% inner regional; 7% outer regional/remote | **Exposure: Employment status - Employed** (≥1 hr paid work/week) vs. Unemployed or not in labour force but wanting to work; those not wanting to work excluded.  **Mental Health Outcome: Mental Health Inventory** (MHI-5) subscale from the Short Form-36 (SF-36) general health scale. | People with a consistent disability status had, on average, mental health scores that were 4.8 points lower (95% CI −7.4 to −2.2) than those without disability. Most of this difference (3.9 points; 95% CI −6.7 to −1.1) was not explained by employment status. About 0.9 points of the difference (95% CI −1.5 to −0.3) were explained by the impact of disability on : employment, meaning that employment accounted for around 19% of the overall effect of disability on mental health. | Employment status  *(Individual)* |
| Ye L, et al. 2023 (11)  Australia-wide  Rurality: not stated | “… investigates the relationship between part-time and full-time employment and mental health for people with and without disability, as well as differences in the relationship by age and sex.” (Abstract, Pg. 1) | Disability as a composite group, compared to those without. | **Design:** Longitudinal cohort study  **Data source:** *Household, Income and Labour Dynamics in Australia (HILDA) Survey*  **Timeframe:** 5 annual waves (2015–2019)  **Analysis:** Fixed effects regression models. | *n* = 2,867 (28.6%) without disability and *n* = 450 (21.3%) with disability, aged 15–24 years. | ***Exposure:* Employment,** categorised as part-time employment, full-time employment, and unemployment, with people who were not in the labour force (those who were neither employed nor unemployed) excluded.  ***Mental Health Outcome:* Mental Health Inventory** (MHI-5) subscale from the Short Form-36 (SF-36) general health scale. | “Among people with disability aged 15–24 years, part-time and full-time employment was associated with average increases in MHI-5 scores of 5.8-points (95% CI 3.0, 8.6) and 4.8-points (95% CI 1.6, 8.0) respectively, compared to when they were unemployed” (Pg. 4)  Excluding psychological disability reduced sample size and slightly reduced effect sizes, but patterns remained consistent. | Employment (both part-time and fulltime vs unemployed)  *(individual)* |

Notes: ACT = Australian Capital Territory; NSW = New South Wales; QLD = Queensland; VIC = Victoria; WA = Western Australia

**References**

1. Foley KR, Bourke J, Einfeld SL, Tonge BJ, Jacoby P, Leonard H. Patterns of depressive symptoms and social relating behaviors differ over time from other behavioral domains for young people with Down syndrome. Medicine (Baltimore). 2015 May;94(19):e710. doi: 10.1097/MD.0000000000000710.
2. Foley KR, Taffe J, Bourke J, Einfeld SL, Tonge BJ, Trollor J, Leonard H. Young People with Intellectual Disability Transitioning to Adulthood: Do Behaviour Trajectories Differ in Those with and without Down Syndrome? PLoS One. 2016 Jul 8;11(7):e0157667. doi: 10.1371/journal.pone.0157667.
3. Jiang B, Walstab J, Reid SM, Davis E, Reddihough D. Quality of life in young adults with cerebral palsy. Disabil Health J. 2016 Oct;9(4):673-81. doi: 10.1016/j.dhjo.2016.04.006. Epub 2016 May 3.
4. King T, Aitken Z, Milner A, Emerson E, Priest N, Karahalios A, Kavanagh A, Blakely T. To what extent is the association between disability and mental health in adolescents mediated by bullying? A causal mediation analysis. Int J Epidemiol. 2018 Oct 1;47(5):1402-1413. doi: 10.1093/ije/dyy154. Erratum in: Int J Epidemiol. 2019 Jun 1;48(3):1025. doi: 10.1093/ije/dyz102.
5. Milbourn B, Mahoney N, Trimboli C, Hoey C, Cordier R, Buchanan A, Wilson NJ. "Just one of the guys" An application of the Occupational Wellbeing framework to graduates of a Men's Shed Program for young unemployed adult males with intellectual disability. Aust Occup Ther J. 2020 Apr;67(2):121-130. doi: 10.1111/1440-1630.12630. Epub 2019 Nov 26.
6. O'Connor M, Jones SC, Gordon C, Joosten A. Exploring Environmental Barriers and Facilitators to Inclusion on a University Campus for Autistic Students. Autism Adulthood. 2024 Mar 1;6(1):36-46. doi: 10.1089/aut.2022.0053. Epub 2024 Feb 28.
7. Olivier E, Azarnia P, Morin AJS, Houle SA, Dubé C, Tracey D, Maïano C. The moderating role of teacher-student relationships on the association between peer victimization and depression in students with intellectual disabilities. Res Dev Disabil. 2020 Mar;98:103572. doi: 10.1016/j.ridd.2020.103572. Epub 2020 Jan 16.
8. Ostojic K, Sharp NL, Paget SP, Morrow AM. Lived experiences of pain in children and young people with cerebral palsy. Dev Med Child Neurol. 2022 Mar;64(3):364-371. doi: 10.1111/dmcn.15061. Epub 2021 Sep 23.
9. Saggers B. Student perceptions: Improving the educational experiences of high school students on the autism spectrum. Improving Schools. 2015;18(1):35-45.
10. Shields M, Spittal MJ, Aitken Z, Dimov S, Kavanagh A, King TL. Does employment status mediate the association between disability status and mental health among young adults? Evidence from the Household, Income and Labour Dynamics in Australia (HILDA) survey. Occup Environ Med. 2023 Sep;80(9):498-505. doi: 10.1136/oemed-2023-108853. Epub 2023 Jul 18.
11. Ye L, Kavanagh A, Petrie D, Dickinson H, Aitken Z. Part-time versus full-time employment and mental health for people with and without disability. SSM Popul Health. 2023 Jun 7;23:101446. doi: 10.1016/j.ssmph.2023.101446.
